# Treatment of aneurysms in the splenic and renal arteries in a single operation: case report and review

**DOI:** 10.1590/1677-5449.200004

**Published:** 2020-06-12

**Authors:** Sergio Quilici Belczak

**Affiliations:** 1 Centro Universitário São Camilo – CUSC, Departamento de Cirurgia Vascular, São Paulo, SP, Brasil.; 2 Instituto de Aprimoramento e Pesquisa em Angiorradiologia e Cirurgia Endovascular – IAPACE, São Paulo, SP, Brasil.

**Keywords:** endovascular procedures, embolization therapy, aneurysm

## Abstract

Visceral and renal artery aneurysms are rare (0.01 to 2%) and their risk of rupture varies between different types and depending on their anatomy and patient context (comorbidities, pregnancy, and liver transplant history). Mortality due to rupture of these aneurysms is around 25%. New techniques and materials derived from neurointervention seem to be promising options for treatment of these aneurysms. In this context, we report the case of a patient undergoing endovascular treatment of both splenic artery and renal artery aneurysms during the same procedure, using Solitaire stents and controlled release coils in both repairs. The patient recovered well with both aneurysms adequately treated. In conclusion, endovascular treatment of splenic and renal artery aneurysms during the same operation is feasible and has proved safe and effective in the case reported.

## INTRODUCTION

Visceral and renal artery aneurysms (VRAAs) are considered rare, with an approximate incidence of 0.01 to 2% of the population.^1^ However, a 10.4% incidence of splenic aneurysms was observed in autopsy studies.^2^

Treatment of VRAAs can be conducted using either open or endovascular techniques. There is a tendency to use endovascular treatment because of its lower morbidity and, more recently, to use materials that are normally used to treat cerebral aneurysms, which has brought countless advantages, such as smaller profiles and greater flexibility and navigability of devices with lower rates of complications.^3^^-^7 It remains a challenge to define which cases should be treated and which should be monitored and nowadays knowledge related to new materials and techniques should be an influential factor in this decision. Against this background, and with the patient’s consent, we report a case in which treatment of aneurysms involving the renal and splenic arteries was accomplished in a single operation, and supplement it with a review of the literature on the subject.

## CASE DESCRIPTION

**Figure 1:**
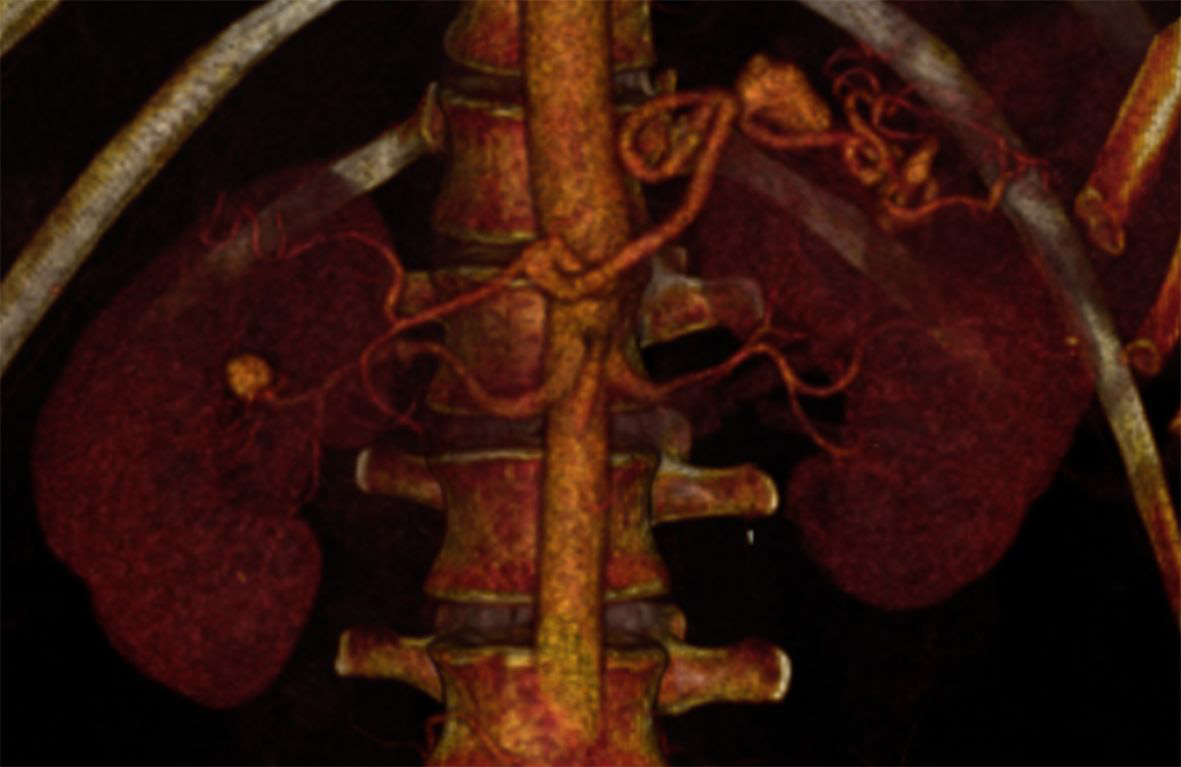
Angiotomography reconstruction showing aneurysms involving the splenic and renal arteries.

The patient was a 34-year-old female who had never been pregnant but was planning to become pregnant the following year. During investigation of suspected kidney stones, a wide-necked saccular aneurysm of the splenic artery measuring 2.8 cm and a saccular aneurysm of the renal artery measuring 1.9 cm were identified (Figure 1). Faced with concomitant aneurysms in both the splenic and renal arteries, fibromuscular dysplasia etiology was suspected and, because of the diameter and asymmetrical saccular morphology of the aneurysms, surgical endovascular treatment was recommended. The patient underwent endovascular repair of both aneurysms during the same surgical operation. Initially, right femoral access was obtained with a 5F introducer and then a cobra catheter was used to catheterize the splenic artery. This access was used to advance the microcatheter and then, initially, the Solitaire® stent (Medtronic, Minneapolis, USA) was deployed, fixing it distally to the artery and proximally to the aneurysm neck. A PX Slim® 2.6 Fr (Penumbra) microcatheter was advanced through the mesh of the stent and, once its location in the aneurysm sac had been confirmed, it was used to perform embolization with Ruby® (Penumbra, Alameda, USA) controlled release coils. Finally, the microcatheter was tractioned to conduct control angiography, which showed patency of the vessel treated, perfusion of the organ, and embolization of the aneurysm (Figures 222C). The same sequence was repeated to treat the renal artery aneurysm (Figures 333C), comprising a total operation duration of 150 minutes to treat both aneurysms (Figure 4). The procedure was conducted in a hemodynamics room (equipped with a Philips Allura FD20 X-ray system) and a total of 48 mL of contrast was used. The patient recovered well, with renal function unimpaired, and was discharged on the following day with double platelet antiaggregation. Control Doppler ultrasound conducted after 1 week showed exclusion of both aneurysms, patency of the vessel treated, and adequate perfusion of the organs (Figures 55B). The patient has been in outpatients follow-up for 90 days and remains asymptomatic.

**Figure 2:**
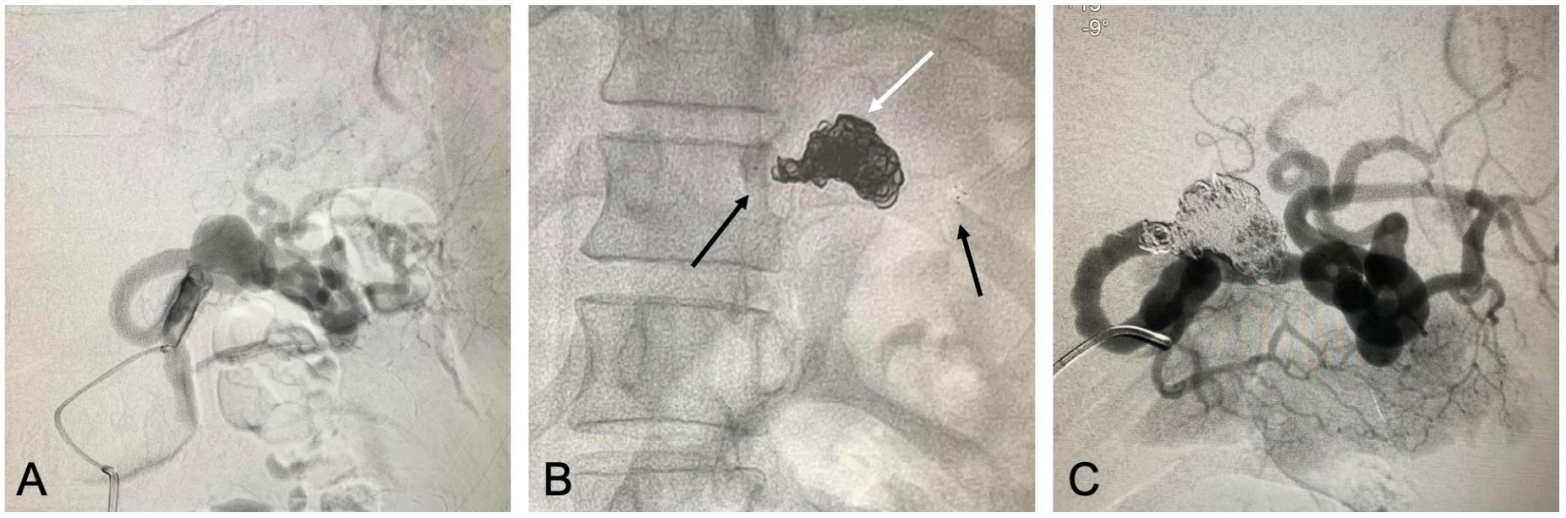
Images of endovascular treatment of the splenic artery aneurysm. (A) Initial arteriography; (B) Image showing the Solitaire® stent well-located and fixed distally to the artery and proximally to the aneurysm neck (black arrows) and Ruby® controlled release coils in the aneurysm interior (white arrow); (C) Control angiography showing exclusion of the aneurysm and patency of the splenic artery.

**Figure 3:**
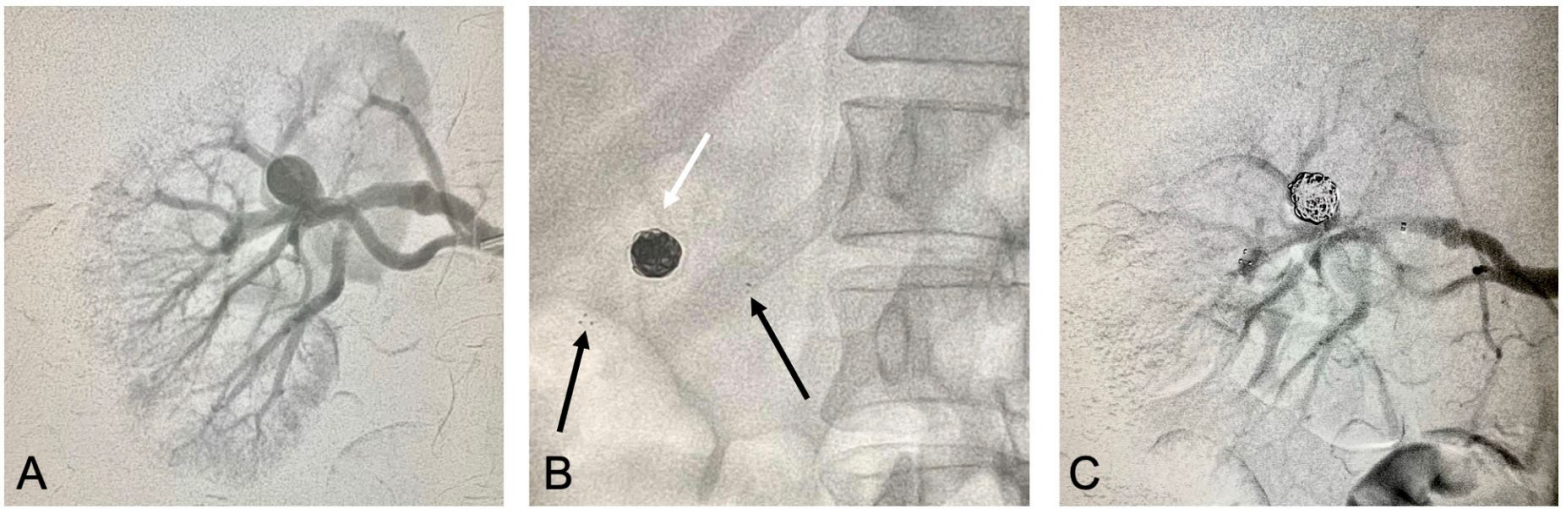
Images of endovascular treatment of the renal artery aneurysm. (A) Initial arteriography; (B) Image showing the Solitaire® stent well-located and fixed distally to the artery and proximally to the aneurysm neck (black arrows) and Ruby® controlled release coils in the aneurysm interior (white arrow); (C) Control angiography showing exclusion of the aneurysm and patency of the renal artery.

**Figure 4:**
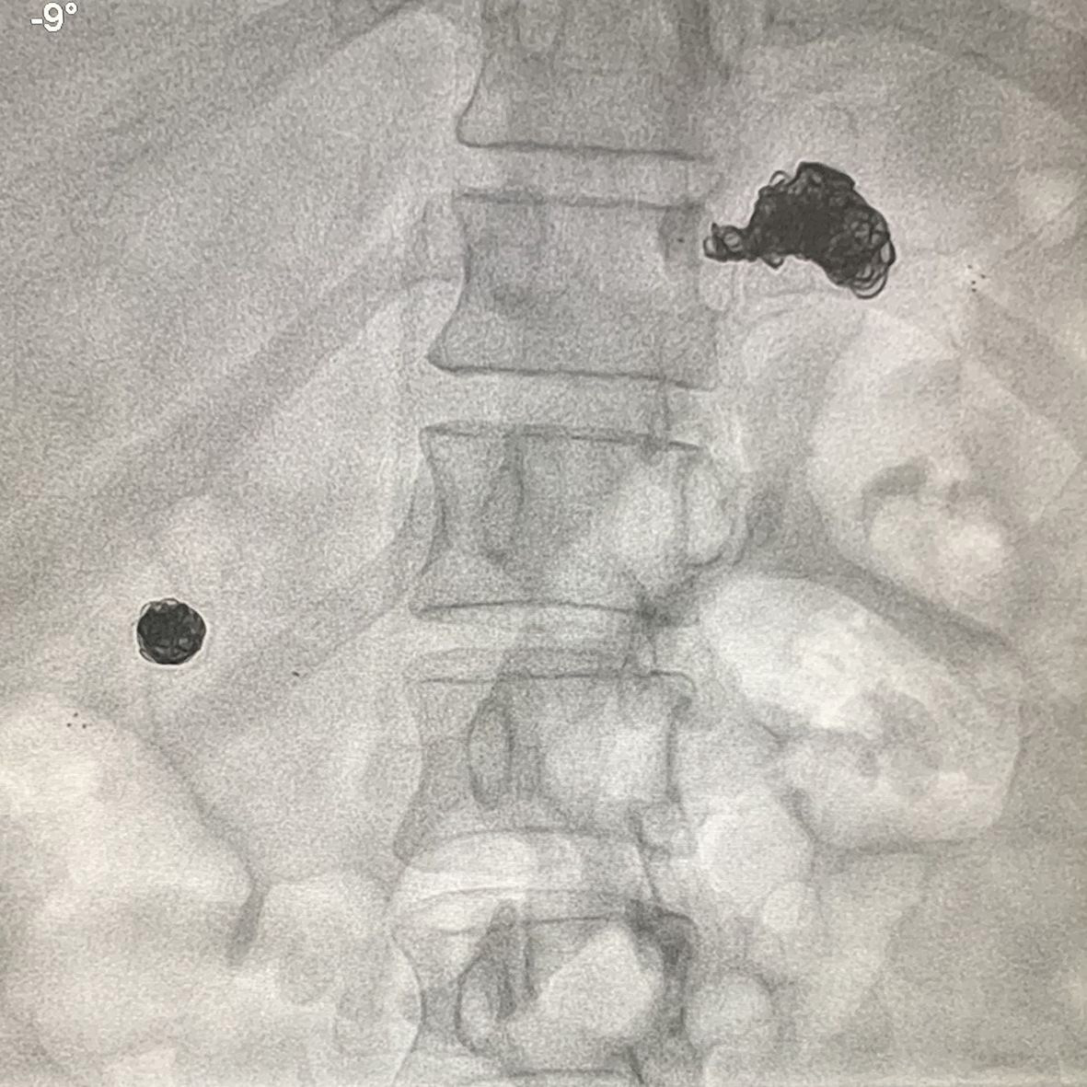
Fluoroscopy image at the end of the procedure showing coils and stents in the left hypochondrium and right flank.

**Figure 5:**
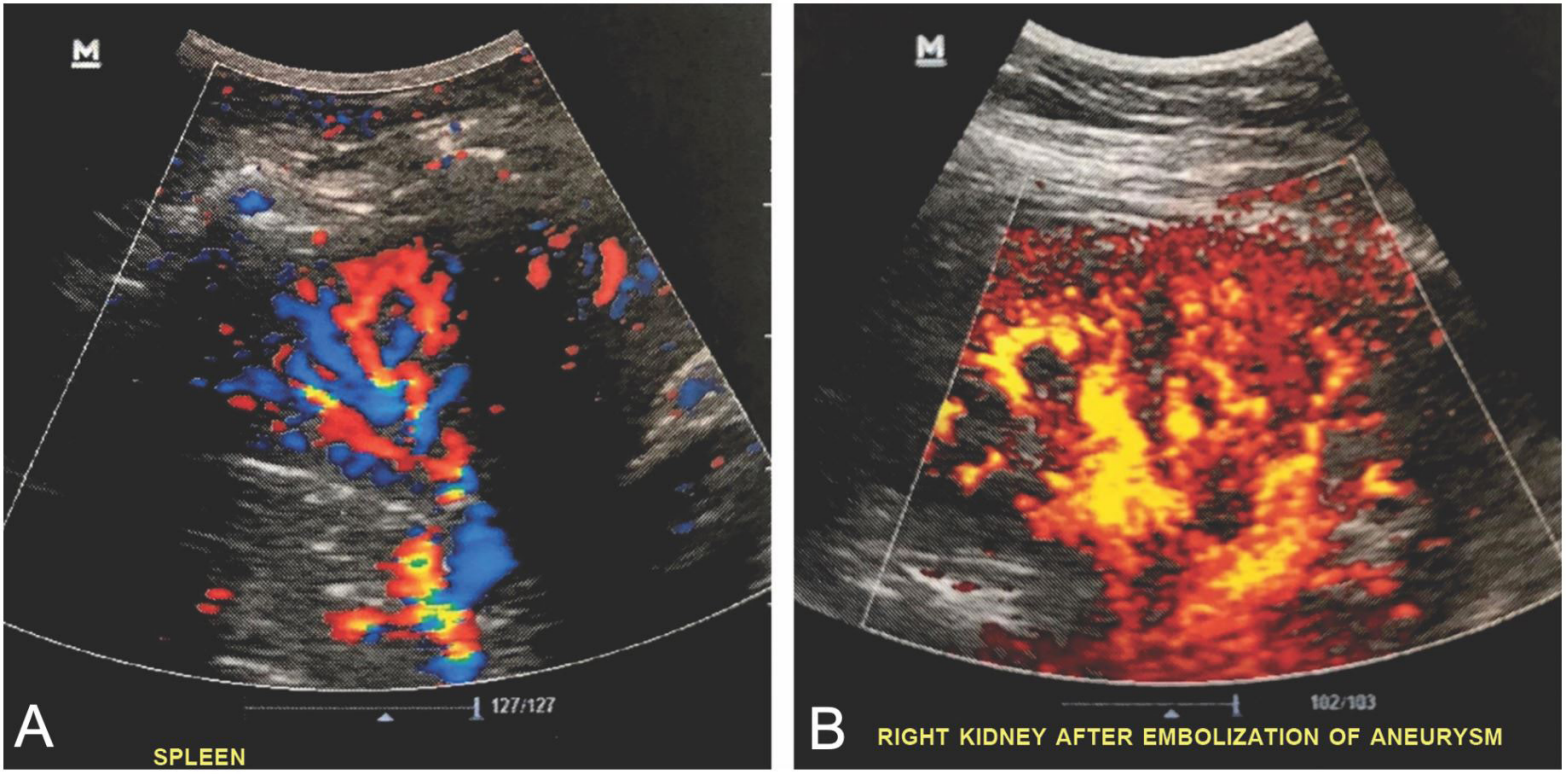
(A) Postoperative control Doppler ultrasonography images showing good perfusion of the splenic parenchyma; (B) Postoperative control Doppler ultrasonography images showing good perfusion of the renal parenchyma.

## DISCUSSION

Visceral and renal artery aneurysms are rare and their natural history has not yet been completely understood. Studies have found evidence of higher prevalence among female patients, which is probably because fibromuscular dysplasia is an important cause in many cases of renal and splenic aneurysms.^7^^,^^8^ The first description of AAVR was written by Beaussiers, in 1770, after finding an aneurysm of the splenic artery during an autopsy. In 1971, Quincke described the classic triad of jaundice, biliary colic and gastrointestinal bleeding, caused by rupture of a hepatic aneurysm. Kehr conducted the first successful surgical treatment of a hepatic aneurysm with ligature, in 1903.^3^^,^^4^

As use of computed tomography and magnetic resonance has grown, incidental findings of AAVR have increased considerably. The majority are asymptomatic or have nonspecific symptomology, which makes early diagnosis less likely. However, in some cases, a range of symptoms can occur, depending on the site of the aneurysms. Mortality from AAVR rupture is approximately 25%, but there are reports that it varies depending on the vessel involved.^5^ The risk of rupture of these aneurysms depends on their size, the speed at which they are growing, and the patient’s comorbidities. For example, it is known that patients with a history of liver transplantation or of pregnancy are at elevated risk of rupture of splenic aneurysms.^6^ It should also be borne in mind that the great majority of these aneurysms are saccular, which puts them at greater risk of rupture, and that the diameters of these vessels reduce in diameter distally, so the same diameter can have a greater risk of rupture at different sites in these vessels.^1^^,^^5^

Indications for treatment include diameter exceeding 2 cm or evidence of growth. Presence of symptoms or complications of AAVR, such as arterial thrombosis or visceral infarction, may also indicate a need for treatment. Similarly, pregnancy and history of liver transplantation should also be considered, particularly in patients with splenic aneurysms.

Several endovascular techniques have been described for treatment of these aneurysms and the choice depends on the characteristics of the aneurysms, the patient’s vascular anatomy, operator experience, and the technology available.^7^^-^9 Morphology, size, diameter of the neck, aneurysm site, organs involved, and presence of downstream branches are determinant factors of which endovascular strategy should be employed.^9^^,^^10^

Saccular aneurysms with narrow necks (proportion aneurysm sac:neck > 2) are candidates for primary embolization of the aneurysm sac with coils or liquid embolic agents.^8^ Saccular aneurysms with wide necks are assigned to techniques for remodeling the neck with the aid of stents or balloons to perform embolization of the aneurysm sac with coils or liquid agents.^10^^-^12 Although described in the literature, we did not conduct embolization with liquid embolization agents in any of the cases in this series. Using stents originally employed for neurointervention procedures, such as the Solitaire® and Lvis®, offers great navigability and flexibility, passing through microcatheters. The Solitaire® also offers the great advantage that it can be repositioned even after it has been fully released. However, more studies of the long-term results of use of these stents in AAVR are needed.^7^ We were unable to find any reports in the literature on treatment of aneurysms in both the splenic and renal arteries during the same surgical operation using these devices.

Covered stents have classically been described for treatment of aneurysms. However they are rarely feasible in bifurcations or when the aneurysm has several downstream branches. The need for a 15 mm landing zone and the rigidity and difficulty of navigation of their deployment systems limit their use.^13^ Another concern is the rate of occlusion of these stents, with reported incidence of up to 17%.^14^

Recent technological advances involve endovascular techniques using flow-modulating stents. These stents have multiple layers specifically designed to reduce the velocity of flow in the interior of the aneurysm sac, provoking thrombosis and maintaining the flow through the principal artery and its branches. These stents are widely used in neurointervention, but there is still little evidence on their use in peripheral vessels, with small case series and reports.^15^^-^17

There is no consensus protocol for follow-up of these aneurysms treated using endovascular techniques. Angiotomography can provide considerable information, but creation of artifacts generated by the metals used to make coils and stents can interfere with viewing. Furthermore, in this context, Doppler ultrasonography conducted by an experienced physician can provide additional information, for example, on flow in the interior and distal to the stent and on whether there is any residual flow in the interior of the aneurysm sac.^18^^,^^19^

Overall, endovascular treatment for AAVR appears to be a good alternative to open treatment. In the case described here, using stents and coils to treat both aneurysms proved effective during treatment and over short-term follow-up. All techniques have their particular characteristics, with advantages and disadvantages, and which technique to employ should be decided on a case-by-case basis. Use of new techniques and materials adopted from neurointervention appears to be a promising options for complex aneurysms with large necks and downstream branches, but prospective, randomized, multicenter studies are needed. In conclusion, endovascular treatment of aneurysms of the splenic artery and the renal artery during the same surgical operation is possible and proved its safety and efficacy in the case reported here.

## References

[1] Pasha SF, Gloviczki P, Stanson AW, Kamath PS (2007). Splanchnic artery aneurysms. Mayo Clin Proc.

[2] Bedford PD, Lodge B (1960). Aneurysm of the splenic artery. Gut.

[3] Pulli R, Dorigo W, Troisi N, Pratesi G, Innocenti AA, Pratesi C (2008). Surgical treatment of visceral artery aneurysms: a 25-year
experience. J Vasc Surg.

[4] Stanley JC, Wakefield TW, Graham LM, Whitehouse WM, Zelenock GB, Lindenauer SM (1986). Clinical importance and management of splanchnic artery
aneurysms. J Vasc Surg.

[5] Shukla AJ, Eid R, Fish L (2015). Contemporary outcomes of intact and ruptured visceral artery
aneurysms. J Vasc Surg.

[6] Abbas MA, Stone WM, Fowl RJ (2002). Splenic artery aneurysms: two decades experience at Mayo
clinic. Ann Vasc Surg.

[7] Bracale UM, Narese D, Ficarelli I (2017). Stent-assisted detachable coil embolization of wide-necked renal
artery aneurysms. Diagn Interv Radiol.

[8] Nosher JL, Chung J, Brevetti LS, Graham AM, Siegel RL (2006). Visceral and renal artery aneurysms: a pictorial essay on endovascular
therapy. Radiographics.

[9] Etezadi V, Gandhi RT, Benenati JF (2011). Endovascular treatment of visceral and renal artery
aneurysms. J Vasc Interv Radiol.

[10] Elaassar O, Auriol J, Marquez R, Tall P, Rousseau H, Joffre F (2011). Endovascular techniques for the treatment of renal artery
aneurysms. Cardiovasc Intervent Radiol.

[11] Dorigo W, Pulli R, Azas L (2016). Early and intermediate results of elective endovascular treatment of
true visceral artery aneurysms. Ann Vasc Surg.

[12] Ferrero E, Ferri M, Viazzo A (2011). Visceral artery aneurysms, an experience on 32 cases in a single
center: treatment from surgery to multilayer stent. Ann Vasc Surg.

[13] Künzle S, Glenck M, Puippe G, Schadde E, Mayer D, Pfammatter T (2013). Stent-graft repairs of visceral and renal artery aneurysms are
effective and result in long-term patency. J Vasc Interv Radiol.

[14] Ierardi AM, Kehagias E, Piffaretti G (2016). ePTFE stent graft in non-steno-occlusive arterial disease: 2 centers
retrospective study. Radiol Med.

[15] Meyer C, Verrel F, Weyer G, Wilhelm K (2011). Endovascular management of complex renal artery aneurysms using the
multilayer stent. Cardiovasc Intervent Radiol.

[16] Henry M, Polydorou A, Frid N (2008). Treatment of renal artery aneurysm with the multilayer
stent. J Endovasc Ther.

[17] Wojtassek M (2013). Managing visceral artery aneurysms. Endovascular Today.

[18] Stelzner C, Abolmaali N, Hecker U, Schellong S (2017). Imaging of visceral vessels. Internist.

[19] Ghariani MZ, Georg Y, Ramirez C (2013). Long-term results of surgical treatment of aneurysms of digestive
arteries. Ann Vasc Surg.

