# Supplementary material for: Treatment of aneurysms in the splenic and renal arteries in a single operation: case report and review
Source: J Vasc Bras. 2020 Jun 12;19:e20200004. doi: 10.1590/1677-5449.200004 (PMC8202182; doi:10.1590/1677-5449.200004)
Supplement: Translation

# Translation: Tratamento de aneurismas de artéria esplênica e renal no mesmo tempo operatório: relato de caso e revisão

**Authors:** Sergio Quilici Belczak

**Affiliations:** 1 Centro Universitário São Camilo – CUSC, Departamento de Cirurgia Vascular, São Paulo, SP, Brasil.; 2 Instituto de Aprimoramento e Pesquisa em Angiorradiologia e Cirurgia Endovascular – IAPACE, São Paulo, SP, Brasil.

**Keywords:** procedimentos endovasculares, embolização terapêutica, aneurisma

## Abstract

Aneurismas de artérias viscerais e renais são raros (0,01 a 2%) e seu risco de ruptura varia entre os diferentes tipos e de acordo com sua anatomia e contexto do paciente (comorbidades, gravidez e histórico de transplante hepático). A mortalidade decorrente da ruptura desses aneurismas é em torno de 25%. Novas técnicas e materiais derivados da neurointervenção parecem alternativas promissoras para o tratamento desses aneurismas. Neste contexto, relatamos um caso de paciente submetida a tratamento endovascular no mesmo procedimento de aneurisma de artéria esplênica e de artéria renal com a utilização de stent Solitaire® (Medtronic, Minneapolis, EUA) e molas de liberação controlada Ruby® (Penumbra, Alameda, EUA). A paciente apresentou boa evolução com ambos aneurismas tratados de forma adequada. Em conclusão, o tratamento endovascular de aneurismas de artéria esplênica e renal no mesmo tempo operatório é exequível e demonstrou segurança e efetividade no caso relatado.

## INTRODUÇÃO

Os aneurismas de artérias viscerais e renais (AAVRs) são considerados raros, com uma incidência aproximada em 0,01 a 2% da população^1^. Entretanto, em estudos de autópsias, evidenciou-se a incidência de aneurismas esplênicos em 10,4%^2^.

O tratamento dos AAVRs pode ser realizado tanto de forma aberta como endovascular. Atualmente há uma tendência a realizar a terapêutica endovascular em decorrência de sua menor morbidade e, mais recentemente, em utilizar materiais que são usualmente empregados no tratamento de aneurismas cerebrais, o que vem conferindo inúmeras vantagens, como diminuição do perfil dos materiais e maior flexibilidade e navegabilidade dos dispositivos com baixas taxas de complicações^3^^-^7. Definir quais casos devem ser tratados e quais devem ser acompanhados ainda permanece um desafio, e atualmente os conhecimentos relacionados a novos materiais e técnicas devem ser fatores influentes nessa decisão. Neste contexto, com consentimento da paciente, relatamos um caso de tratamento no mesmo ato cirúrgico de aneurismas de artérias renal e esplênica, acompanhado de uma revisão de literatura sobre o tema.

## DESCRIÇÃO DO CASO

Paciente do sexo feminino, de 34 anos, nuligesta, com planejamento de engravidar no próximo ano e em investigação de suspeita de urolitíase identificou um aneurisma sacular de colo largo de artéria esplênica de 2,8 cm e um aneurisma sacular de artéria renal de 1,9 cm (Figura 1). Frente à presença de aneurisma concomitante em artéria esplênica e artéria renal, suspeitou-se de etiologia de displasia fibromuscular e, em decorrência do diâmetro e morfologia sacular assimétrica dos aneurismas, indicou-se tratamento cirúrgico mediante terapia endovascular. A paciente, então, foi submetida a correção endovascular de ambos aneurismas no mesmo ato cirúrgico. Inicialmente, realizou-se um único acesso femoral direito com introdutor 5F e, com cateter cobra, a artéria esplênica foi cateterizada. Através desse acesso, passou-se o microcateter e inicialmente o stent Solitaire® (Medtronic, Minneapolis, EUA) foi implantado, fixando-se distalmente à artéria e proximalmente ao colo do aneurisma. Através da malha do stent, passou-se o microcateter PX Slim® 2.6 Fr (Penumbra, Alameda, EUA) e, confirmada pela angiografia a localização do microcateter no saco do aneurisma, procedeu-se à embolização com molas de liberação controlada Ruby® (Penumbra, Alameda, EUA). Finalmente, tracionou-se o microcateter para realização de angiografia de controle evidenciando perviedade do vaso tratado, perfusão do órgão e embolização do aneurisma (Figura 222C). A mesma sequência foi realizada para o tratamento do aneurisma de artéria renal (Figura 333C), compondo um total de 150 minutos de procedimento para tratamento de ambos aneurismas (Figura 4). O procedimento foi realizado em hemodinâmica (equipamento Philips Allura FD20), sendo utilizado, no total, 48 mL de contraste. A paciente apresentou boa evolução, permanecendo com função renal inalterada e recebendo alta no dia seguinte com dupla antiagregação plaquetária. Foi realizado Doppler de controle 1 semana depois, evidenciando exclusão dos aneurismas, perviedade do vaso tratado e adequada perfusão dos órgãos (Figura 55B). A paciente encontra-se em acompanhamento ambulatorial há 90 dias e permanece assintomática.

**Figura 1:** Reconstrução de angiotomografia evidenciando aneurisma em artéria esplênica e artéria renal.

**Figura 2:** Imagens do tratamento endovascular do aneurisma de artéria esplênica. (A) Arteriografia inicial; (B) Imagem evidenciando stent Solitaire® bem alocado fixando-se distalmente à artéria e proximalmente ao colo do aneurisma (setas pretas) e molas de liberação controladas Ruby® no interior do aneurisma (seta branca); (C) Angiografia de controle evidenciando exclusão do aneurisma e perviedade da artéria esplênica.

**Figura 3:** Imagens do tratamento endovascular do aneurisma de artéria renal. (A) Arteriografia inicial; (B) Imagem evidenciando stent Solitaire® bem alocado, fixando-se distalmente na artéria e proximalmente ao colo do aneurisma (setas pretas) e molas de liberação controladas Ruby® no interior do aneurisma (seta branca); (C) Angiografia de controle evidenciando exclusão do aneurisma e perviedade da artéria renal.

**Figura 4:** Imagem de fluoroscopia ao final do procedimento evidenciando as molas e os stents em hipocôndrio esquerdo e flanco direito, respectivamente.

**Figura 5:** (A) Imagens de ultrassonografia Doppler de controle pós-operatório, evidenciando boa perfusão do parênquima esplênico; (B) Imagens de ultrassonografia Doppler de controle pós-operatório, evidenciando boa perfusão do parênquima renal.

## DISCUSSÃO

Os AAVRs são raros e sua história natural ainda não é completamente compreendida. Estudos evidenciam prevalência maior em pacientes do sexo feminino, o que deve ser decorrente da displasia fibromuscular ser causa importante em muitos casos de aneurismas renais e esplênicos^7^^,^^8^. A primeira descrição de AAVR foi feita por Beaussiers em 1770, quando encontrou um aneurisma de artéria esplênica em uma autópsia. Em 1971, Quincke descreveu a clássica tríade de icterícia, cólica biliar e sangramento gastrointestinal, causada pela ruptura de um aneurisma hepático. Kehr realizou o primeiro tratamento cirúrgico bem-sucedido de um aneurisma hepático com ligadura em 1903^3^^,^^4^.

Com a crescente realização de tomografia computadorizada e ressonância magnética, o achado incidental de AAVR aumentou muito. A maioria são assintomáticos ou com sintomatologia inespecífica, o que dificulta o diagnóstico precoce. Porém, em alguns casos, diferentes sintomas podem ocorrer, dependendo da localização dos aneurismas. A mortalidade decorrente da ruptura de AAVR é aproximadamente 25%; entretanto, há relatos de que ela varia de acordo com o vaso acometido^5^. O risco de ruptura desses aneurismas depende do seu tamanho, da sua velocidade de crescimento e das comorbidades do paciente. Sabe-se, por exemplo, que, em pacientes com histórico de transplante hepático ou gestação, há risco elevado de ruptura de aneurismas esplênicos^6^. Deve-se considerar também que a grande maioria desses aneurismas é sacular, o que lhes confere maior risco de ruptura, e que estes vasos vão diminuindo os diâmetros em suas porções mais distais de tal forma que o mesmo diâmetro confere maior risco de ruptura em diferentes localizações nesses vasos^1^^,^^5^.

As indicações para o tratamento incluem diâmetro maior que 2 cm ou evidência de crescimento. A presença de sintomas ou complicações de AAVR, como trombose arterial e infarto visceral, podem ser indicativos de tratamento. Da mesma forma, gestação e histórico de transplante hepático, em especial em pacientes portadores de aneurismas esplênicos, devem ser considerados.

Diversas técnicas endovasculares foram descritas para o tratamento desses aneurismas, e a escolha depende das características dos aneurismas, anatomia vascular do paciente, experiência do operador e tecnologia disponível^7^^-^9. Morfologia, tamanho, diâmetro do colo, localização do aneurisma, órgão afetado e presença de ramos eferentes são fatores determinantes de qual estratégia endovascular deverá ser realizada^9^^,^^10^.

Os aneurismas saculares com colo estreito (proporção saco aneurismático:colo > 2) são candidatos para embolização primária do saco aneurismático com molas ou agentes embólicos líquidos^8^. Os aneurismas saculares com colo largo são destinados para técnicas de remodelamento do colo assistida por stent ou balão para embolização com molas ou agentes líquidos do saco aneurismático^10^^-^12. Apesar de descrita na literatura, não realizamos em nenhum caso desta série a embolização com agentes embolizantes líquidos. A utilização de stents, empregados primariamente em procedimentos de neurointervenção, como o Solitaire® e o Lvis®, apresenta grande navegabilidade e flexibilidade, passando no interior de microcateteres. Inclusive, o Solitaire® apresenta a grande vantagem de poder ser reposicionado mesmo após ter sido completamente liberado. Porém, mais estudos dos resultados a longo prazo da utilização desses stents em AAVR são necessários^7^. Não encontramos nenhum relato na literatura sobre o tratamento no mesmo ato cirúrgico de aneurisma de artéria esplênica e renal com esses dispositivos.

Stents revestidos foram classicamente descritos para o tratamento dos aneurismas; entretanto, eles dificilmente podem ser utilizados em bifurcações ou na presença de mais ramos eferentes do aneurisma. A necessidade de uma zona de ancoragem de 15 mm e a rigidez e falta de navegabilidade do seu sistema de entrega limitam o seu uso^13^. Outra preocupação é a taxa de oclusão desses stents com uma incidência relatada de até 17%^14^.

Os avanços tecnológicos recentes envolvem técnicas endovasculares com stents moduladores de fluxo. Esses stents apresentam múltiplas camadas especificamente desenhadas para reduzir a velocidade de fluxo no interior do saco aneurismático, promovendo trombose e manutenção do fluxo na artéria principal e seus ramos. Eles são amplamente utilizados na neurointervenção, mas sua utilização em vasos periféricos ainda carece de evidências, com pequenas séries e relatos^15^^-^17.

Em relação ao acompanhamento desses aneurismas tratados por técnica endovascular, não há nenhum protocolo consensual. A angiotomografia pode fornecer diversas informações, mas a geração de artefatos decorrente dos metais inerentes às molas e stents pode dificultar a visualização. Além disso, neste contexto, a realização de ultrassonografia Doppler com médico experiente pode acrescentar informações, por exemplo, o fluxo no interior e distalmente ao stent e se há algum fluxo no interior do saco aneurismático^18^^,^^19^.

Assim, o tratamento endovascular de AAVR com técnicas endovasculares parece ser boa alternativa ao tratamento aberto. No caso apresentado, a utilização de stent com molas para tratamento de ambos aneurismas se mostrou efetiva no tratamento e na evolução a curto prazo. Todas as técnicas apresentam suas particularidades, com vantagens e desvantagens, e a indicação da técnica utilizada deve ser individualizada. A utilização de novas técnicas e materiais derivados da neurointervenção parece ser uma alternativa promissora para aneurismas complexos com colos largos e ramos eferentes, porém mais estudos multicêntricos prospectivos e randomizados são necessários. Em conclusão, o tratamento endovascular de aneurismas de artéria esplênica e renal no mesmo tempo operatório é exequível e demonstrou segurança e efetividade no caso relatado.
